# An economic framework for assessing the impact of domestic mining policies on affordable energy transition

**DOI:** 10.1038/s41598-024-64535-1

**Published:** 2024-06-13

**Authors:** Mahelet G. Fikru

**Affiliations:** 1 Department of Economics, Missouri University of Science and Technology, 500 West 13th Street, Rolla, MO 65409 USA; 2 Thomas J. O’Keefe Institute for Sustainable Supply of Strategic Minerals, Missouri University of Science and Technology, Rolla, MO 65409 USA

**Keywords:** Critical minerals, Electric vehicles, Mining policy, US, Economic models, Governance, Environmental economics

## Abstract

The global transition towards sustainable energy sources necessitates a delicate balance between incentivizing domestic mineral inputs and ensuring affordable energy transition. This paper investigates a diverse set of policies aimed at promoting domestic mining and their implications for achieving an affordable energy transition. Through a comprehensive economic framework, we analyze the effects of demand and supply-side policies on import reliance, production costs, and the overall progress of the energy transition. By examining various scenarios and their outcomes, we provide insights into the potential challenges and opportunities associated with designing mineral policies that facilitate both domestic mining growth and affordable clean energy technologies. Our findings highlight the importance of striking a balance between promoting domestic resources and ensuring affordability in the pursuit of a sustainable energy future.

## Introduction

Critical minerals and metals are non-fuel materials vital for a nation’s economy, national security, and energy transition goals^1^. Most critical minerals and metals are hard-to-replace inputs in producing energy transition technologies such as batteries for electric vehicles (EV) and films used in photovoltaic (PV) solar cells. Examples of energy transition minerals and metals include cobalt, graphite, lithium, manganese, nickel, rare earth elements (REEs), and others. Nate et al.^2^ present a method for identifying minerals important for the green energy transition and find that cobalt, graphite, and lithium are among the ones with the lowest availability and highest predicted demand for energy applications. In addition, several other minerals that are not necessarily on the critical minerals list (such as copper and iron) are also needed in the manufacturing of *energy transition technologies (ETTs)*^2^. For example, compared to conventional power sources such as coal and natural gas, producing energy from solar panels requires 115% and 132% more copper per megawatt of power, respectively^3^.

**Figure 1:**
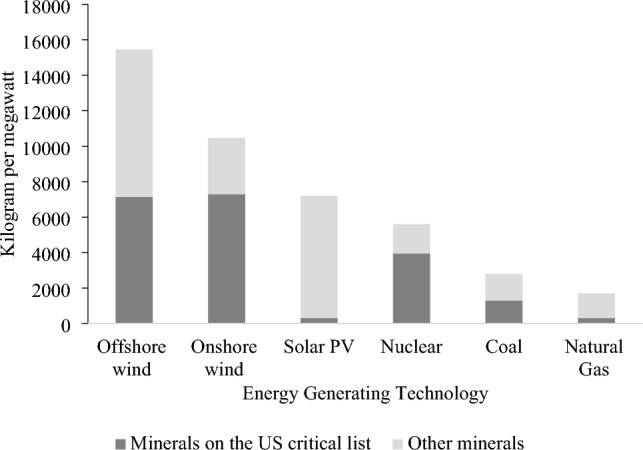
Minerals needed for energy transition technologies are either on the critical list (nickel, manganese, cobalt, chromium, zinc, and REEs) or not (copper, molybdenum, silicon, and others). Source: Author’s reformulation based on data from^3^.

Figure 1 presents the mineral intensity of several energy-generating technologies illustrating the high mineral intensity for renewable/cleaner energy technologies than fossil-based technologies, whether these minerals are on a nation’s critical list or not. Besides energy-generating technologies, ETTs such as electric vehicles (EVs) require up to six times more minerals than conventional vehicles due to the high mineral intensity of EV batteries. According to estimates by the International Energy Agency (IEA), the global demand for energy transition minerals (including lithium, cobalt, and nickel) would reach 7 million tons to produce wind turbines, solar panels, EV batteries, and other ETTs^4^.

Given the high mineral intensity of ETTs^5^, governments and nations worldwide are increasingly recognizing the strategic importance of encouraging *domestic mining and local mineral processing* (referred to as *onshoring*) to bolster economic resilience and resource security^6^. The motivation stems from a multifaceted perspective, encompassing economic, geopolitical, and environmental considerations^7,8^. For example, domestic mining can help nations reduce dependence on imports, thereby enhancing national security and insulating economic systems from global supply chain disruptions. Furthermore, incentivizing local mining could contribute to job creation, economic growth, and technological innovation. Domestic sourcing or onshoring could also help ensure a reliable supply chain while also addressing environmental concerns through adherence to stringent domestic regulations^9^. Mineral onshoring strategies often involve implementing financial incentives, regulatory frameworks, and infrastructure investments to support and attract domestic mineral extraction and local metal refining operations.

While the strategic move towards encouraging domestic mining is gaining momentum, there are notable research gaps in understanding the full spectrum of impacts that such policies (that is, strategies used for onshoring) may have. One research gap is understanding how and to what extent domestic mining policies could affect downstream markets where ETTs are produced. Despite several studies discussing the geopolitical aspects of mineral onshoring^7,8^, there are not many studies examining the interplay between mineral and mining policies in upstream markets and the resultant effects on affordable energy transition in downstream markets. As countries continue to prioritize domestic mining and encourage local mineral sourcing, understanding the economic mechanisms and channels governing the relationship between policies in domestic mineral and metal markets and their implications on downstream industries becomes paramount. This study addresses the literature gap by comprehensively examining the economic channels through which policies instituted in upstream minerals and metals markets impact downstream markets, where ETTs are produced. This is done by developing a generic economic model to address the following research question: How can domestic mining policies (that is, policies instituted in upstream markets) be designed to allow for a more affordable energy transition system?

The study adopts a comprehensive economic framework to evaluate the influence of domestic mining policies on the cost of the energy transition. The methodological approach aligns with the economic principles of supply and demand. It considers how changes in production costs, influenced by factors such as raw material prices, can impact market dynamics, specifically the quantity of ETT supplied and the price consumers pay per unit. Firstly, the study conceptualizes the market for domestic mineral and metal inputs (DMMIs) which involves modeling the dynamics of supply and demand for domestic minerals and metals crucial for energy transition technologies, such as electric vehicle (EV) batteries and solar panels. The initial market equilibrium, characterized by high import reliance, serves as the foundational baseline. Subsequently, the study delves into analyzing various supply-side policies aimed at augmenting domestic mineral supply. These policies encompass financial incentives directed towards domestic mining, refining, and processing, to reduce the marginal cost associated with domestic mineral production. Concurrently, the study also evaluates demand-side policies geared toward stimulating the demand for domestic minerals. These policies encompass measures like import taxes on minerals and subsidies for ETT manufacturers utilizing domestic inputs, all designed to curtail import reliance. Furthermore, the study explores combined policy scenarios, amalgamating supply and demand policies to gauge their collective impact on domestic mineral prices and import reliance.

Secondly, the study investigates the impact of different mining policy scenarios on the production cost of ETTs where policy-induced changes in DMMI prices could affect ETT supply potentially causing a change in the price, and hence the affordability, of ETTs. The model is interpreted under the assumption that DMMIs (or inputs) are the main cost drivers for the manufacturing of ETTs where a decline in the cost of procuring DMMIs (due to policy incentives) could translate into higher volumes of ETT production at a lower price per unit (facilitating affordability, that is, ETT consumers could benefit from lower ETT prices), and vice versa. The study draws conclusions that can be used in designing mineral and mining policies that facilitate an affordable energy transition, balancing the goals of reducing import reliance and fostering the growth of the clean energy sector.

Section “Upstream policies shaping outcomes in downstream markets: identification of economic channels” presents an economic model that is used to examine the impact of domestic mining policies on ETT markets. Section “Implications for policy making: the US case” discusses the case of US mining policies in light of the proposed model framework while Section “Conclusion” presents a summary of recommendations and questions for future studies.

## Upstream policies shaping outcomes in downstream markets: identification of economic channels

There is a growing number of studies on the topic of mineral-energy nexus where most studies examine the impact of energy transition indicators (e.g., renewable capacity) on mineral trade dynamics^10^. Several of these studies find that decarbonization requires more mineral resources some of which are geographically concentrated, hence increasing import reliance for countries that do not have mineral deposits^11,12^. For example, Yu et al.^13^ find an increase in mineral and metal resource demand as renewable energy technologies are developed. Likewise, Fikru and Kilinic-Ata^12^ show that as nations continue to invest in ETTs, such as renewable energy and electrification, they are expected to increase their mineral import demand. Beylot et al.^14^ estimate copper and aluminum quantities needed for achieving France’s energy transition goals by 2050. Another example is^15^ which examines the impact of renewable generation on mineral market returns.

Srivastava and Kumar^16^ conducted a comprehensive literature review on studies that are at the intersection of energy transition and mineral resources and concluded that while there is a growing number of studies examining geopolitical and global trade aspects of the mineral-energy nexus^7,8,17^, there are very limited studies with a policy perspective. Bazilian^18^ examines the critical role of minerals in achieving energy transition and recommends policy and regulatory responses to reduce bottlenecks in accessing key inputs. Such responses include resource mapping, technology research, and developing financial instruments in commodity markets to mitigate risks related to market dynamics. However, it is still not well understood whether and under what conditions/scenarios such policies would affect progress in energy transition. It is also not clearly understood under what conditions upstream mining policies could facilitate an affordable energy transition.

The following sub-sections present a generic economic framework with several scenarios (supply-side policies, demand-side policies, and a combination of both) to shed light on the intricate dynamics between upstream mineral policies and downstream ETT markets, providing valuable insights for policymakers, researchers, and industry stakeholders. Each scenario explores how policies affect the cost of production, import reliance, and overall progress in the ETT market. First, the market for domestic mineral and metal inputs (DMMIs) is presented followed by the market for ETTs. The model only captures domestic inputs and so do not directly capture international trade dynamics, comparative advantages, and competition issues.

### Market for domestic minerals and metals

**Figure 2:**
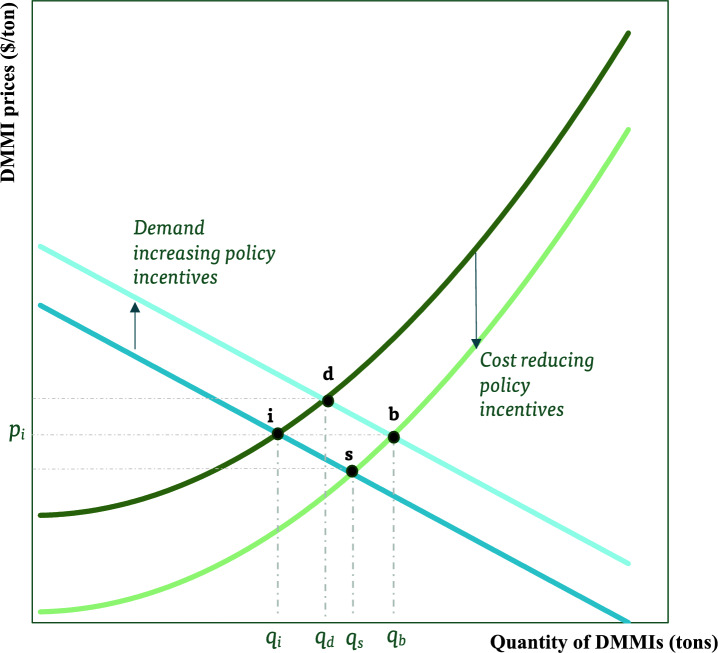
How policy incentives affect domestic minerals and metals (Source: Author’s elaboration).

The domestic market for mineral and metal inputs crucial to ETTs is depicted in Fig. 2. The equilibrium marked as point “*i”* symbolizes the initial state where import reliance is high. The bundled quantity of domestic mineral and metal inputs (DMMI) used in ETTs is presented on the x-axis. For example, a given volume of bundled DMMIs could include cobalt, lithium, and nickel used in EV batteries. The DMMI market is represented by a combination of demand and supply forces where the supply curve represents the sum of quantity supplied by all domestic mining companies at the indicated marginal opportunity cost (e.g., dollar per ton of minerals). Demand is assumed to originate from ETT producers operating locally (e.g., EV battery makers, solar panel producers, etc.) who also purchase a certain portion of their mineral and metal requirements from overseas (i.e., import reliance).

The initial market equilibrium (point “*i*”) corresponds to the quantity of domestic minerals and metals, $$q_{i}$$, at the indicated per unit price ($$p_{i} )$$. At this initial equilibrium, the import reliance is assumed to be high. In other words, the amount $$q_{i}$$ is low relative to total minerals and metals needed for the ETT. For example, in the US net import reliance, defined as a percent of apparent consumption, for cobalt is 76%, lithium is 25%, and nickel is 48% yielding an overall average of close to 50% for the given bundle of the three inputs^19^.

Policy-induced changes in both supply and demand are considered independently and then collectively to assess their impact on reducing import reliance. The goal of each of these policies is primarily to decrease import reliance by increasing the volume of DMMIs. This implies that the policy-maker’s primary goal in the DMMI market (upstream market) is to replace imported inputs with domestically sourced inputs, and not primarily motivated by energy transition goals. The goal is also not to increase total mineral demand to produce a higher level of ETTs (e.g., mineral intensity may not change). Overall, this requires improving the comparative advantage of the country in mineral and metal production and providing preferential treatment (e.g., financial incentives) for producers that use domestic and not imported inputs.

Table 1 outlines examples of policy tools targeting upstream (that is, DMMI) markets, categorizing them into cost-reducing policies to increase supply and policies increasing demand for domestic inputs. The policy goal is to encourage domestic mining, processing, and manufacturing while reducing reliance on imports. The policy tools could increase demand/supply for domestic inputs. First, domestic supply can be enhanced by using *cost-reducing financial incentives* provided directly for mining companies engaged in the extraction of mineral ores within the country (e.g., mining sites in Alaska could extract more ores containing critical minerals with lower costs) or companies engaged in the processing and refining of these ores to produce inputs for ETTs (e.g., smelters that produce germanium from ores and concentrates could increase capacity with lower costs). The vertical distance between the two supply lines indicates, the per unit cost to the policymaker, of subsidizing domestic mining and processing.

**Table 1:** Example of policies instituted on upstream markets (Source: Author’s elaboration).

Market forces (agents)	Supply of domestic minerals and metals (local mines and processing facilities)	Demand for domestic minerals and metals (EV battery producers)
Policy	Cost-reducing policies increase domestic supply	Policies increasing demand for domestic inputs
Example of policy tools	Financial incentives for domestic mineral supply chains including permitting, exploration, refining, production (e.g., tax credits, grants, loans), and subsidizing innovative extraction and processing technologies	Import tax or restriction on ores and/or metals; export tax on ores; financial incentives for ETT manufacturers with domestic sourcing
Goal	Encourage domestic mining, processing, and manufacturing of mineral and metal inputs	Encourage domestic sourcing of mineral and metal inputs
Outcome	Reduce import reliance	Reduce import reliance
Impact on downstream industries	Per unit cost of mineral procurement declines ($p < p_{i}$)	Per unit cost of mineral procurement increases ($p > p_{i}$)

Such policy incentives are cost-reducing because they provide direct or indirect subsidies (e.g., per unit of minerals, or as a percent of minerals processed, provide support for mineral exploration, and allow efficient permitting) that lower the marginal opportunity cost of producing minerals domestically. If policy incentives are only instituted on supply and not demand, then the cost of ETT production declines with more minerals/metals sourced domestically and import reliance declines. In this scenario, presented by equilibrium ‘*s*’, total demand for mineral inputs (which is the sum of domestic and imported sources) does not necessarily increase, keeping other factors constant. Policies instituted on the supply side can achieve the goal of reducing import reliance at a lower cost per unit for downstream industries.

Second, the demand for DMMIs could increase if policy incentives are designed so that ETT producers demand relatively more domestic inputs by reducing their import reliance. The vertical distance between the two demand lines indicates the per unit cost of subsidizing the consumption of domestic minerals and metals. Examples of tools could be an import tax which raises the cost of imported materials to be higher than DMMIs (e.g., assuming imported and domestic inputs are technically substitutable), or a tax credit for domestic sourcing (e.g., EV battery makers receiving a subsidy for domestic sourcing). If policies induce an increase in demand while supply is the same/fixed, then the cost of procuring DMMI will increase per ton while domestic sourcing increases and import reliance declines. In this scenario, presented by equilibrium ‘*d*’, total demand for minerals (which is the sum of domestic and imported sources) may not necessarily increase. However, such type of policies might end up increasing the cost of downstream industries (e.g., per unit cost of domestic minerals increases compared to the initial equilibrium but is still relatively cheaper than importing minerals) and increasing the cost of domestic sourcing.

If policies are instituted on both demand and supply, then the net impact would be to increase the quantity of DMMIs used in ETTs as presented by the equilibrium “*b*”. In this scenario, producers of ETTs reduce their mineral import reliance by a larger amount where the impact on their per unit costs could either change or stay the same as presented in Fig. 2. In either scenario, ETT producers will find it relatively cheaper to use domestic sources relative to importing minerals due to a combination of policies increasing both demand and supply. To illustrate, suppose the average price of a ton of imported *bundle* of mineral/metal input is $95 per ton at the initial equilibrium (“*i*”) while the average price of the same bundle from local sources is $100 per ton giving rise to an initial equilibrium (say, “*i*” with high import reliance where $$p_{i} = 100$$). With supply-side policies in isolation, the average domestic price could decline (say to $90/ton) while demand-side policies in isolation could increase domestic price (say to $103/ton) while at the same time increasing import price say to $105/ton (domestic mineral is more expensive than before (*i*) but cheaper than imports).

A combination of both demand and supply-increasing policies could lead to a much larger reduction in import reliance where domestic mineral prices stay the same as before, increase or decline (e.g., within the range of $90–$105 per ton from the previous example) depending on the relative magnitudes of incremental changes in the demand and supply (e.g., stronger cost-reducing effects on supply would push the price of domestic minerals to the lower range while a stronger demand-increasing policy could push the price of domestic minerals to the higher range).

Overall, domestic mineral/mining policies could end up changing the initial mineral (that is, DMMI) price and this price change can be transmitted to the ETT market in the form of a change in the per unit cost of mineral procurement. For each of these three scenarios ($p = p_{i}$, $p < p_{i}$, and $p > p_{i}$,) and the corresponding equilibria marked in Fig. 1 (*b*, *s*, *d*), we discuss the potential channels through which the domestic mining policies presented in Table 1 affect the market for ETTs.

### Market for energy transition technologies

**Figure 3:**
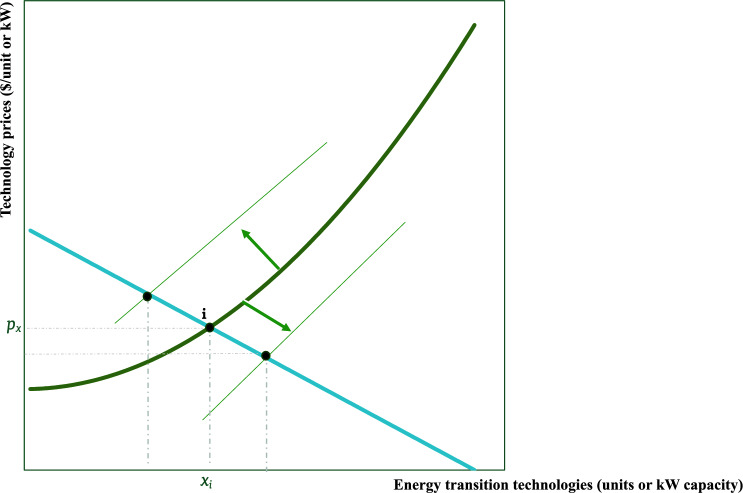
The impact of domestic mining policies on ETT markets (Source: Author’s elaboration).

The ETT market (e.g., EV batteries) is represented in Fig. 3 where suppliers are producers of the technology and buyers are consumers of these technologies (e.g., households who buy EVs, EV makers who buy the batteries to make EVs, buyers of solar panels, etc.). The initial equilibrium is presented as “*i*’ which determines the quantity of technology bought and sold, say $x_{i}$ (e.g., number of EVs bought/sold) at the given price ($p_{x}$). The initial equilibrium marked as “*i*” establishes the baseline for technology production and consumption. Subsequent policy-induced changes in the price of domestic minerals and metals (caused by upstream policies) directly influence the cost of producing ETTs, presenting varying outcomes for the ETT market. This is under the assumption that mineral procurement takes a significant portion of the cost of producing ETTs. After the policies presented in Table 1 are instituted, if the price of DMMIs increases, say $p > p_{i}$, (corresponding to equilibrium *d*) this directly increases the marginal cost of producing the ETT (supply shifts to the left). If say $p < p_{i}$, (corresponding to equilibrium *s*) this would reduce the marginal cost of producing the ETT (supply shifts to the right). Otherwise, the supply curve stays the same (for example, corresponding to equilibrium *b*).

In the first scenario, the average ETT maker experiences a higher domestic mineral price than the initial equilibrium, but domestic minerals remain competitive relative to foreign ones due to the impact of the instituted (demand-side) policy. The price of the ETT increases while the quantity traded declines. This scenario implies that the mineral policy instituted in the upstream market created a hurdle for energy transition goals by disincentivizing the now costly energy transition even when facilitating domestic sourcing. This implies that the demand-increasing policy instituted in isolation on the market for DMMIs achieves its goal of reducing import reliance but at the cost of slowing down the energy transition, and at the cost of making energy transition less affordable.

In the second scenario, where the cost-reducing effect of domestic mining policies is strong enough, the cost of production for the average ETT maker reduces thereby increasing supply which ultimately translates to a lower cost of ETT and a higher equilibrium level of energy transition. In this case, it could be possible to achieve domestic sourcing along with an increase in energy transition at a lower cost. In the final scenario, there is no impact on the ETT market, and hence domestic mineral policies do not impact progress in the energy transition while achieving a lower rate of import reliance.

**Figure 4:**
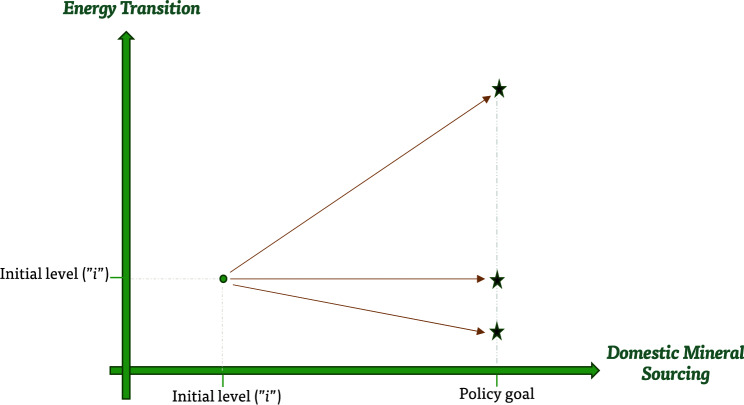
The impact of domestic mineral sourcing policies on energy transition (Source: Author’s elaboration).

The results from the different scenarios are summarized in Fig. 4 and imply that domestic mining policies that preferentially favor DMMIs (over imports) could have three potential implications for energy transition: (1) increasing energy transition and reducing the cost of transition (affordability) through their impact on mineral/metal procurement costs, (2) declining energy transition while increasing the cost of transition, or (3) not changing the initial level or cost of energy transition. The first option materializes if upstream domestic mining policies prioritize supply-side policies (e.g., financial incentives for the mining industry to reduce costs) while the second option occurs if mining policies prioritize demand-side policies (e.g., encouraging domestic mineral sourcing). The third option materializes with a balancing act of demand and supply side policy tools.

## Implications for policy making: the US case

Following Executive Order 13,817 on December 20, 2017, the US Geological Survey (USGS) identified a list of 35 nonfuel minerals as critical based on an assessment of their importance for the US economy and national security. The criticality of these minerals is measured by three metrics: the extent of the nation’s import reliance, supply chain vulnerability, and disruption potential. This list was updated in 2022 based on the most up-to-date data and criticality evaluation criteria. Currently, there are 50 critical minerals including 16 rare earth elements (REEs)^20,21^.

**Figure 5:**
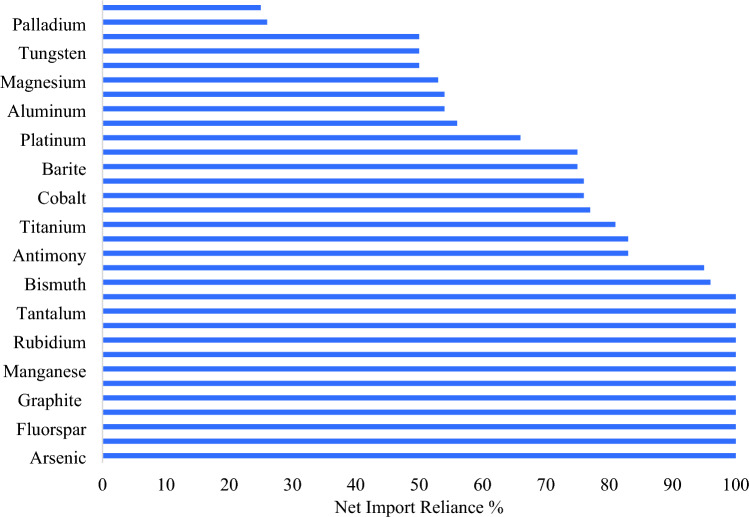
US net import reliance as a percent of apparent consumption for a sample of 33 critical minerals (Source: Author’s reformulation based on data compiled from^19^).

The United States, despite being among one of the top countries with substantial reserves of minerals, faces significant import reliance on key ETT inputs. For instance, the US remains 100% import-reliant on 12 of the 50 minerals in the critical minerals list, and over 50% import-reliant on other 31 minerals^19,22^. Figure 5 summarizes net import reliance for a sample of critical minerals with the highest net imports. When reserves are available, some of the ores and concentrates containing metals may be exported overseas for further processing and refining. For example, the US exports large amounts of nickel ore and concentrate to Canada due to insufficient domestic processing capacity^23^. For minerals such as graphite and manganese, the US has no known domestic reserves and hence limited room for encouraging domestic mining^19^.

### Policy goals in the upstream industry

The current policy approach towards critical minerals centers around addressing the three metrics based on which minerals are assessed for their criticality and these are high import reliance, supply chain vulnerability, and disruption potential. High import reliance is addressed by investing in domestic mining and processing capabilities to decrease import dependency. Supply chain vulnerability and disruption potential is addressed by implementing strategies to diversify international supply chains from extraction to end-use including recycling of metals and materials, reducing mineral intensities, or use of alternative (e.g., non-critical) inputs. Disruption potential is also addressed via a combination of risk mitigation strategies such as stockpiling and building material buffers^19^.

In this study, we focus on those policy tools with the primary goal of strengthening the domestic supply chain of energy transition minerals by expanding domestic mining and mineral/metal production. We also summarize policy tools that seek to strengthen supply chain resiliency by reducing vulnerability and supply disruption risk. Table 2 presents a summary of these policy tools along with implications for DMMI and ETT markets based on the conceptual discussions presented in section “Upstream policies shaping outcomes in downstream markets: identification of economic channels”. [Note: The current US policy approach also emphasizes responsible mining which includes adherence to environmental, social, and labor principles^20^ as well as community engagement, and tribal consultation^21,22,24,25^. The implications of these are not discussed in this study.]

**Table 2:** Summary of tools used to achieve policy goals in the US (Source: Author’s elaboration based on^25–33^.

Policy goal	Reduce import reliance	Reduce vulnerability/supply disruption risk and strengthen supply chain resiliency
Implications	Encourage domestic mining, processing, and manufacturing of critical minerals and metals (onshoring or switching mineral sources from overseas to local)	1. Diversify sources of minerals and metals 2. Reuse and recycle materials 3. Develop substitute inputs (alternatives) or reduce mineral use via innovations
Current tools	Grants, loans, and other financial incentives for mineral extraction/processing and related R&D; improved permitting; developing workforce; providing resources for advanced mapping in explorations^25,26^	1. Friend shoring; consumer tax credits based on local/regional mineral sourcing (IRA)^30,33^; new trade agreements/partnerships^27^ 2. Financial incentives for recycling/reprocessing batteries^32^, or related R&D; recycling or non-traditional sources of minerals^29^ 3. Financial incentives for mineral substitution technologies^31^ or related R&D^28^
Impact on DMMI markets	Increase in supply of domestic minerals/metals (cost-reducing effects on mining/processing)	DMMI supply/demand may not change with #1; DMMI demand may decline with #2 and #3. A decline in DMMI demand reduces cost of procuring domestic minerals (e.g., ${p<p}_{i}$), thereby increasing ETT supply with a lower price per unit
Impact in ETT markets	ETT supply could increase facilitating energy affordable energy transition if (1) mineral input cost is a significant portion of production costs, (2) DMMI supply shift translates into a significant reduction in domestic mineral price, or (3) ETT supply is sufficiently responsive to reduced (domestic) mineral prices	Switching import partners may change cost of ETT production; using alternative/recycled inputs may alter cost of ETT production

#### Reducing import reliance as a policy goal

Since the 2017 Executive Order 13,817, the US mineral policy has emphasized strengthening the domestic supply chain for critical minerals. Addressing this goal would require, among others, the development of new mining sites, new mineral exploration projects, and a significant increase in mineral processing capacity using advanced technologies^21^. The policy goal emphasizes reducing import dependencies, expanding domestic mining and mineral/metal production including processing and refining, modernizing mining laws and regulatory frameworks^23^, and developing research and workforce.

The majority of US policy tools are supply-side policies which take the form of subsidies, tax credits, and grants to mining businesses. Hence these could act as cost-reducing policies in the upstream market. Key examples of such policies are (1) grants, subsidies, tax incentives, and loans for specific mining projects including R&D, (2) geological mapping resources, and (3) opening federal lands. Such policy incentives, when instituted in isolation, could have the potential to act as cost-reducing policies as presented by the equilibrium labeled as ‘*s*’ in Fig. 2.

#### Reducing vulnerability/supply disruption risk and strengthening supply chain resiliency as a policy goal

Besides reducing import dependency through domestic mining and local metal processing, the US mining policy has also emphasized the need to diversify mineral sources through international trade agreements (e.g., friend shoring) to reduce supply disruption risk. This goal can also be achieved by investing in alternative and unconventional methods of increasing supply (e.g., recycling, processing mine wastes, etc.)^21,24,25^, recycling materials, and finding alternatives to mineral inputs.

### Examples of policy incentives in the upstream industry

The Bipartisan Infrastructure Law (BIL) as enacted in 2022 in the Infrastructure Investment and Jobs Act (IIJA Law) provides several fund allocations (approximately $407 million) to support the mining industry in (1) R&D and demonstration of critical minerals extraction, separation and refinery from mine wastes (Section 40205), (2) critical minerals mining and recycling, as well as reclamation strategies and technology development (Section 40210), (3) loan guarantee program through the Department of Energy to increase the supply of domestically produced critical minerals (Section 40401), (4) grant awards for research, supply, processing, and recycling of battery critical minerals and materials (Sections 40207, 40208, 40210).

These incentives come in the form of directly funding projects that potentially extract minerals and metals using advanced technology, and unconventional methods (e.g. waste brine after power generation in geothermal brines can be a source of lithium before cycling back to the ground, recycling, recovery from mine tailings, etc.), and federal loans and grants to the private sector. The purpose of funding research is to lower the cost of extracting minerals from ore or recycled products; to support new processes or technologies to extract and process (to reduce overall production cost), as well as identify potential substitutes (e.g., lower demand for minerals per output like battery)^26,29,30^.

Related to this Section 40201/2 of the Infrastructure Investment and Jobs Act (IIJA of 2021 also known as Bipartisan Infrastructure Law BIL) establishes the Earth Mapping Resources Initiative (Earth MRI) within the USGS to provide mapping resources and collect data on abandoned mine waste sites (appropriations totally $320 million for years 2022–2026). Enhanced geological mapping provides information on potential mineral deposits (including in private lands) and helps target mining efforts accordingly. This means the private industry does not need to incur the cost of mineral exploration and this reduces the industry’s costs and reduces uncertainties regarding the grade and quality, as well as location of deposits.

Section 40206 of the IIJA has additional provisions to increase the speed of permitting and review on federal lands. Increasing mining operations on federal lands (e.g., allowing lithium mining in federal lands in Nevada) encompassing about 712 million acres of the federal mineral estate (30% of total area) and this makes available new mine explorations.

Effective 2023, the Inflation Reduction Act provides a 10% tax credit (percent of the cost of production which includes both mining and refining the mineral and can be staked) to critical minerals producers as part of the new advanced manufacturing production tax credit but with no phase-out in 2030. Tax incentives help enhance domestic mineral production (mining and ore refining) by reducing the tax burden and reducing costs related to capital investments and depreciation.

Finally, the IRA appropriated up to $500 million for the enhanced use of the Defense Production Act (DPA) to help strengthen the US supply chain in critical minerals. In March 2023, President Biden expanded the scope of DPA to support critical minerals used in batteries to onshore the battery supply chain for the energy supply chain (this allows more projects and mineral producers to be eligible to get funding under DPA).

The Energy Act of 2020, The BIL of 2021, the CHIPS and Science Act, and the IRA combined authorized $8.5 billion for critical minerals activities at the DOE and DOI which includes rare earth and critical mineral recovery from unconventional sources, creating consortiums and research facilities, USGS resource assessment (Earth MRI), and workforce development grants. Since the IRA was enacted, over $45 billion in private sector investment has been announced in clean vehicle and battery supply chains across + 75 facilities across the country^34^. This signals the response of industry^35^.

### The impact of downstream policies on upstream markets

The economics literature has examined the response of mineral import demand to the increasing capacity of cleaner energy^12,32^. For instance, Kilinc-Ata et al.^36^ estimate that a 10% expansion in renewable energy capacity is expected to lead to an approximately 9% increase in mineral import demand. Studies also show that the overall demand for energy transition minerals and materials (imported plus available local) will increase with increases in ETT investments. Lee and Glynn^37^ find that to achieve a net-zero goal by 2050, demand for minerals will increase by 20-fold from 2020 consumption levels. Likewise, Hund et al.^5^ show that the deployment of clean energy technologies is expected to increase demand for graphite, lithium, and cobalt by approximately 500% by the year 2050.

In addition to policy incentives instituted in upstream markets, the US has several policy incentives in downstream markets. These policies instituted in ETT markets are consistent with the nation’s decarbonization goals and are expected to increase mineral demand. However, the specific impact on DMMI markets is not well understood. For example, the Bipartisan Infrastructure Law (BIL) as enacted in 2022 in the Infrastructure Investment and Jobs Act (IIJA Law) provides $15 billion to support EV deployment over the next 5 years. About half of this allocation goes to building EV charging stations along highways and within communities, while the other half goes to clean buses and ferries. These initiatives are expected to facilitate the deployment of EVs across the nation and increase demand for domestic production of clean vehicles, putting upward pressure on key minerals and metal inputs needed for EV batteries. Such ETT policy incentives are expected to increase demand for ETT, and while the policy goal is to advance ETTs, as more ETTs are produced demand for mineral/metal inputs is expected to increase as well^12^.

On the other hand, the Inflation Reduction Act of 2022 (IRA) provides a financial incentive (the Clean Vehicle Credit) for increasing *demand* for domestically and regionally extracted and processed minerals. The financial incentive is provided for the ultimate consumer (i.e., buyers of clean vehicles such as new plug-in and fuel cell EVs purchased in 2023 or after) and not producers of ETT. This policy tool provides consumer tax credits of up to $7,500 for clean vehicles made with locally or regionally sourced critical minerals and a partial credit if the domestic requirement on critical minerals is not fulfilled. The two eligibility requirements for receiving the Clean Vehicle Credit are: (1) 40% of the critical mineral value must be extracted, processed, or recycled in the US or a free trade nation [Note: Free trade nations are initially listed but the list could change in the future: Australia, Bahrain, Canada, Chile, Colombia, Costa Rica, Dominican Republic, El Salvador, Guatemala, Honduras, Israel, Jordan, Korea, Mexico, Morocco, Nicaragua, Oman, Panama, Peru, Singapore, and Japan]. This percentage will increase to 50% in 2024, 60% in 2025, 70% in 2026, and 80% after 2026, and (2) 50% or more of the value of the battery must be manufactured or assembled in the US where the percentage requirement increases to 60% in 2024–25, 70% in 2026, and 90% in 2028. Consumers may also benefit from other state-level subsidies for clean vehicles^31^. About two-thirds of EV sales were eligible as of 2022 with more expected to benefit from the provision by adjusting their input and production sourcing.

ETT policy incentives, such as the Clean Vehicle Credit, are intended to create *additional demand* for domestic mineral inputs versus imported ones by increasing demand for ETTs made from domestic inputs^30^. Hence, policies like the $7,500 consumer tax credit could potentially increase demand for domestically produced electric vehicles with domestic mineral content (e.g., increasing demand in Fig. 3). However, these are policies instituted in the EET market (Fig. 3) which are then expected to affect the upstream market (Fig. 2). Thus, assuming Fig. 3 presents the market for domestically produced ETTs that fulfill the domestic sourcing requirement of the IRA, a policy-induced increase in demand will likely cause an increase in ETT production and a price increase for a given supply. As more EVs are produced to fulfill the increase in demand, more units of DMMIs are likely to be demanded. This could potentially cause the demand for DMMIs to increase, and the equilibrium shifts from “*i*” to “*d*”. Finally, the Clean Vehicle Credit is a tax credit provided for ETT consumers and not ETT producers. As a result, the credit could increase demand for eligible ETT but not necessarily the supply.

**Figure 6:**
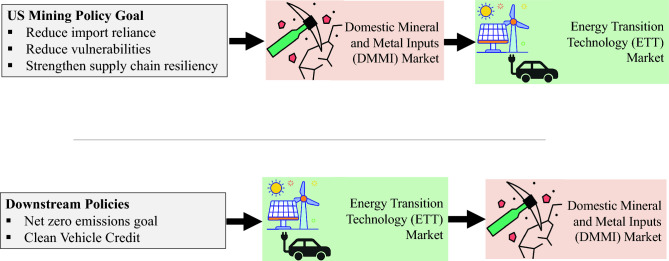
Upstream policies affect ETT markets while downstream policies affect DMMI markets (Source: Author’s own elaboration)*.*

Using the US case discussed in this section, Fig. 6 illustrates the potential impact of upstream policies (supply and/or demand side) on ETT markets and the impact of downstream policies on demand for DMMIs. Generally defined net zero policy goals could increase demand for ETTs while the Clean Vehicle Credit increases demand for locally produced EVs with domestic mineral sourcing. These downstream policies could increase demand for ETTs which in turn induce an increase in demand for mineral and metal inputs, from domestic and international sources as well as preferentially from domestic sources, respectively.

## Conclusion

As nations strive for sustainable energy transition, the intricate relationship between policies incentivizing domestic mining and the affordability of this transition comes to the forefront. This paper explores the balance required to design effective mineral and mining policies that not only encourage domestic sourcing but also ensure the affordability of clean energy technologies. Through a comprehensive economic model, we examine the impact of such policies on the downstream market for energy transition technologies, emphasizing the need for a strategic balancing act. The analysis delves into scenarios of supply and demand-side policies, as well as their combination, shedding light on the implications for import reliance, production costs, and overall progress in the energy transition market. Insights from this study provide valuable guidance for policymakers and stakeholders in achieving a harmonious and sustainable energy transition.

How can domestic mining policies (that is, policies instituted in upstream markets) be designed to allow for a more affordable energy transition system? The analysis presented in this study illustrates the need to strike a balancing act between using demand-side and supply side policies in the domestic minerals market to ensure the cost of energy transition does not change (in particular, increase) with domestic mining and mineral sourcing. If domestic mining policies prioritize supply-side measures, such as offering financial incentives to the mining industry to reduce costs, this could lead to an accelerated energy transition. By ensuring a steady supply of domestically mined minerals, the cost of transitioning to cleaner energy sources may decrease. This approach emphasizes affordability and economic feasibility. Conversely, if mining policies prioritize demand-side actions, such as encouraging domestic mineral sourcing, the energy transition might slow down. While this approach promotes self-reliance (e.g., lower net imports for minerals), it could also raise the cost of energy transition (under the assumption that mineral procurement is a significant share of ETT cost of production). Balancing affordability with self-sufficiency becomes crucial here. These findings highlight the role of economic models in clarifying trade-offs and identifying the potential impacts of domestic mining policies on energy transition markets.

The discussion presented in this study is not without limitations. First, an important aspect of the mineral-energy nexus is the role of trade and environmental policies that could potentially alter both the upstream and downstream markets. Even if this study did not model trade and environmental protection policies, these are important in determining the pace of the energy transition and future models ought to account for the impact of policies other than those targeting domestic mining. More studies are needed to understand how trade and environmental policies interact with domestic mining policies and policies that advance energy transition. Second, the models presented in this study assume that an increase in ETT would lead to an energy transition, that is a replacement of fossil with renewables or cleaner sources, instead of using the added ETT to fulfill increased demand for energy. Hence, the model assumes energy demand is the same which in some contexts may not universally hold (e.g., energy rebound effects). Third, there is a role for demand and supply elasticities in governing the rate of response of market variables to policy instruments, and hence empirical modeling approaches are required to estimate the strengths of the changes predicted in this study. For example, while cost-reducing policies instituted in the DMMI market could lower costs for ETT producers, the rate of reduction in costs may not be statistically or economically significant. Finally, the supply side policies discussed in this study (e.g., those providing financial incentives for the mining industry, etc.) are likely to require substantial government expenditure leading to market distortions (e.g., deadweight loss). Hence, these policies may not be a cost-effective long-term solution as they requires a reallocation of tax revenues to support an industry characterized by its measurable environmental and social impacts.

## Data Availability

The datasets used and/or analyzed during the current study is available from the corresponding author on reasonable request.
